# Myeloperoxidase and Septic Conditions Disrupt Sphingolipid Homeostasis in Murine Brain Capillaries In Vivo and Immortalized Human Brain Endothelial Cells In Vitro

**DOI:** 10.3390/ijms21031143

**Published:** 2020-02-09

**Authors:** Madeleine Goeritzer, Eva Bernhart, Ioanna Plastira, Helga Reicher, Christina Leopold, Thomas O. Eichmann, Gerald Rechberger, Corina T. Madreiter-Sokolowski, Jürgen Prasch, Philipp Eller, Wolfgang F. Graier, Dagmar Kratky, Ernst Malle, Wolfgang Sattler

**Affiliations:** 1 Division of Molecular Biology and Biochemistry, Gottfried Schatz Research Center, Medical University of Graz, Graz 8010, Austria; madeleine.goeritzer@medunigraz.at (M.G.); eva.bernhart@medunigraz.at (E.B.); ioanna.plastira@medunigraz.at (I.P.); helga.reicher@medunigraz.at (H.R.); christina.leopold@medunigraz.at (C.L.); corina.madreiter@hest.ethz.ch (C.T.M.-S.); j.prasch@medunigraz.at (J.P.); wolfgang.graier@medunigraz.at (W.F.G.); dagmar.kratky@medunigraz.at (D.K.); ernst.malle@medunigraz.at (E.M.); 2 BioTechMed-Graz, Graz 8010, Austria; thomas.eichmann@uni-graz.at (T.O.E.); gerald.rechberger@uni-graz.at (G.R.); 3 Institute of Molecular Biosciences, University of Graz, Graz 8010, Austria; 4 Center for Explorative Lipidomics, BioTechMed-Graz, Graz 8010, Austria; 5 Department of Health Sciences and Technology, ETH Zurich, Schwerzenbach 8603, Switzerland; 6 Department of Internal Medicine, Intensive Care Unit, Medical University of Graz, Graz 8036, Austria; philipp.eller@medunigraz.at

**Keywords:** blood–brain barrier, calorimetry, ceramides, cytokines, fatty acid, mitochondrial function, myeloperoxidase, sphingomyelins

## Abstract

During inflammation, activated leukocytes release cytotoxic mediators that compromise blood–brain barrier (BBB) function. Under inflammatory conditions, myeloperoxidase (MPO) is critically involved in inflicting BBB damage. We used genetic and pharmacological approaches to investigate whether MPO induces aberrant lipid homeostasis at the BBB in a murine endotoxemia model. To corroborate findings in a human system we studied the impact of sera from sepsis and non-sepsis patients on brain endothelial cells (hCMEC/D3). In response to endotoxin, the fatty acid, ceramide, and sphingomyelin content of isolated mouse brain capillaries dropped and barrier dysfunction occurred. In mice, genetic deficiency or pharmacological inhibition of MPO abolished these alterations. Studies in metabolic cages revealed increased physical activity and less pronounced sickness behavior of MPO^−/−^ compared to wild-type mice in response to sepsis. In hCMEC/D3 cells, exogenous tumor necrosis factor α (TNFα) potently regulated gene expression of pro-inflammatory cytokines and a set of genes involved in sphingolipid (SL) homeostasis. Notably, treatment of hCMEC/D3 cells with sera from septic patients reduced cellular ceramide concentrations and induced barrier and mitochondrial dysfunction. In summary, our in vivo and in vitro data revealed that inflammatory mediators including MPO, TNFα induce dysfunctional SL homeostasis in brain endothelial cells. Genetic and pharmacological inhibition of MPO attenuated endotoxin-induced alterations in SL homeostasis in vivo, highlighting the potential role of MPO as drug target to treat inflammation-induced brain dysfunction.

## 1. Introduction

The neurovascular unit separates most regions of the brain from the peripheral circulation to maintain the specialized central nervous system (CNS) micromilieu. Brain microvascular endothelial cells (BMVEC) form the morphological basis of the blood–brain barrier (BBB) by formation of tight junction and adherens junction complexes [1]. These junctional complexes inhibit paracellular leakage, maintain CNS homeostasis via polarized expression of transporter systems, and protect most regions of the brain from injuries. However, BBB function is compromised under inflammatory conditions and can aggravate neuronal dysfunction [2]. The lipid composition of BMVEC plays a pivotal role in barrier function by establishing a unique environment that inhibits caveolae vesicle formation to suppress transcytosis and ensure BBB integrity [3,4].

Due to structural and signaling requirements, a functional sphingolipid (SL) rheostat is indispensable for physiological vascular and BBB function [5]. De novo synthesis of SL occurs in the ER where serine palmitoyltransferase (SPT) catalyzes decarboxylation of palmitoyl-CoA and condensation with serine generating 3-ketosphinganine, which is further converted to dihydro -sphingosine, di-hydro ceramide, and finally ceramide (Cer; Figure S1). In the Golgi Cer serves as substrate for sphingomyelin (SM) synthases to generate SM species. The relative levels of these three interconvertible SL metabolites are regulated by sphingosine kinase (SPHK)1 and -2, which phosphorylate sphingosine to produce sphingosine-1-phosphate (S1P) ([6]; Figure S1). S1P is exported and is available as auto- or paracrine signaling molecule via five S1P receptors (S1PR1-5). Of these, S1PR1 plays a central regulatory role in BBB function [7]. Reactive oxygen (e.g., H_2_O_2_) and nitrogen (e.g., ONOO^−^) regulate SL metabolism [8] and signaling pathways [9]. Disruption of SL homeostasis is associated with endothelial barrier dysfunction, a hallmark of sepsis, acute lung injury, and sepsis-associated encephalopathy [5,10,11]. Disruptive BBB dysfunction in a murine lipopolysaccharide (LPS)-induced endotoxemia model is accompanied by alterations of the S1P rheostat in brain capillaries and dysregulation of SL homeostasis in serum and brain [11].

Reactive oxygen species (ROS) are potent regulators of vital physiological functions [12] and may improve systemic defense mechanisms [13]. However, conditions of oxidative stress, defined as an imbalance between pro- and anti-oxidants leading to disruption of redox circuitry and macromolecular damage [14], might trigger peripheral, cardiovascular, metabolic, or neurological pathologies. This discrepancy between beneficial and disease-mediating effects of ROS is probably the underlying cause for the failure of antioxidant intervention trials that were mostly ineffective or detrimental [15]. Based on this limited success, inhibition of ‘ROS toxifiers’ was proposed as interference strategy [16]. Myeloperoxidase (MPO), a component of the armamentarium of the innate immune system, generates ROS and reactive nitrogen species. In the presence of chloride ions, MPO, which is abundantly present in neutrophils and monocytes, converts the relatively weak two electron oxidant H_2_O_2_ to highly reactive hypochlorous acid (HOCl), a prototypic example of a ‘ROS toxifier’ reaction. Chronic activation of phagocytes results in elevated levels of HOCl that can modify a wide range of biomolecules including antioxidants, nucleotides, DNA, (lipo)proteins, and lipids. Thus, prolonged production of HOCl causes tissue injury [17,18] and leads to the formation of a chlorinated lipidome with deleterious functions [18]. In addition to modification of ether-phospholipid plasmalogens [18], HOCl is able to modify SL [19] with a second order rate constant in the range observed for plasmalogens [20].

Here we hypothesized that inflammatory conditions activate MPO and, according to the ‘ROS toxifier’ concept, affect lipid and energy metabolism in brain capillaries or BMVEC due to impaired adaptation to pro-oxidant conditions. We used an LPS-induced murine endotoxemia model (recapitulating some of the sepsis-associated features observed in the mouse cecal ligation puncture model; Ref. [21]) to study the impact of MPO on SL homeostasis in brain capillaries of wild-type (wt) and MPO^−/−^ mice or by pharmacological inhibition of MPO with 4-aminobenzoic acid hydrazide (ABAH). In a human system, we investigated the role of inflammatory mediators (TNFα and serum obtained from sepsis patients) on human brain endothelial cell (hCMEC/D3) function. We investigated the role of inflammatory mediators (TNFα and serum from sepsis patients) in a human system based on the function of human brain endothelial cells (hCMEC/D3). In this ‘humanized’ in vitro system, we studied gene expression of interleukin-6 (*IL-6*) and *TNFα*, and a selected set of metabolic enzymes that regulate SL turnover, barrier and mitochondrial function, SL homeostasis, and cytokine expression in vitro.

## 2. Results

**Figure 1:**
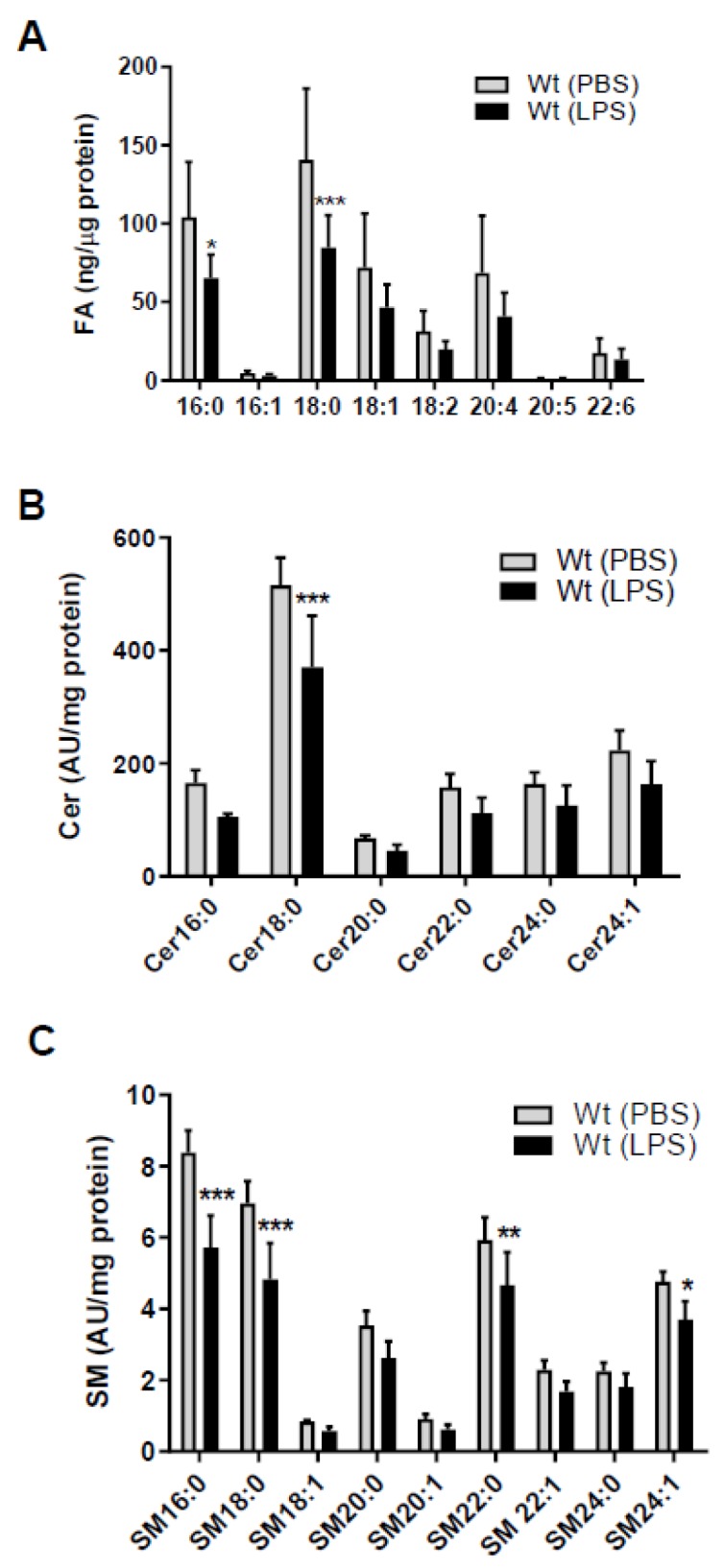
Peripheral lipopolysaccharide (LPS) administration decreases fatty acid (FA), ceramide (Cer), and sphingomyelin (SM) content in brain microcapillaries of wild-type (wt) mice. Wt mice received a single i.p. injection of PBS (200 µL) or LPS in PBS (from *Escherichia coli*, 0111:B4 in PBS, 8.3 µg/g body weight) and were sacrificed 16 h after injection. (**A**) GC analysis of the total FA content of mouse brain microcapillaries (pooled from two brains per sample) as FA methylester derivatives. Significance was calculated by ANOVA, followed by Bonferroni correction. Data are shown as mean (*n* = 6–7) + SD. (**B**) Levels of Cer and (**C**) SM species in isolated brain microcapillaries (pooled from 3 brains per sample) were measured by LC-ESI-MS/MS. Cer and SM species are displayed on basis of their acyl chain composition. The values obtained were normalized to the internal standard and protein amount (arbitrary units (AU)/mg protein). Data are shown as mean (*n* = 5) + SD. Significance was calculated by ANOVA, followed by Bonferroni correction. *, *p* < 0.05; **, *p* ≤ 0.01; ***, *p* ≤ 0.001.

Since SL homeostasis of brain capillaries is a major determinant of BBB function [4,5] we first clarified the impact of sepsis-like conditions on fatty acid (FA), Cer, and SM content of murine brain microcapillaries. Endotoxemia induced by LPS injection markedly reduced palmitic (C16:0) and stearic (C18:0) acid concentrations (Figure 1A) in wt mice. There was also a tendency (though statistically not significant) for a reduction in other FA species (C16:1, C18:1, C18:2, C20:4, and C22:6). As palmitoyl-CoA is an essential substrate for the rate-limiting step in de novo SL synthesis we next determined Cer and SM content of brain capillaries by LC-ESI-MS/MS. Among the Cer species (with dihydroxy(d)18:1 backbone) Cer18:0 was significantly reduced (27%) in response to systemic LPS-induced inflammation (Figure 1B). Among the SM-d18:1 species, SM16:0, 18:0, 22:0, and 24:1 were significantly reduced as compared to control mice (Figure 1C). We also found a decrease for other Cer and SM species, however, statistically not significant (Figure 1B,C). SL metabolism and results are summarized in Figure S1.

**Figure 2:**
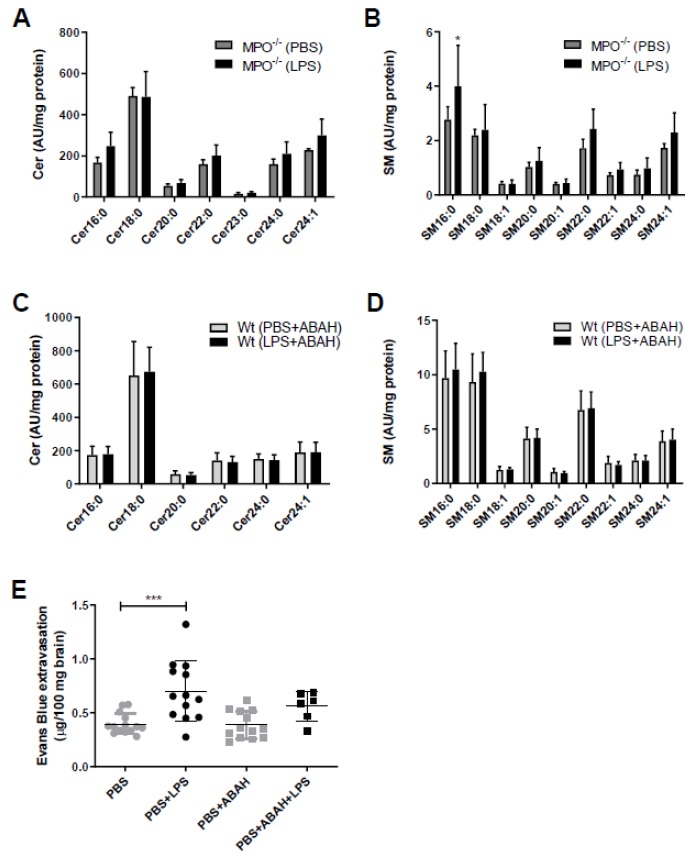
Myeloperoxidase (MPO)^−/−^ or pharmacological MPO inhibition by ABAH prevents aberrant brain capillary sphingolipid homeostasis in response to peripheral LPS. (**A**,**B**) Cer and SM content of microcapillaries (pooled from three brains per sample) of PBS or LPS (8.3 µg/g body weight) injected MPO^−/−^ mice were analyzed by LC-ESI-MS/MS. The values were normalized to the internal standard and protein amount. Data are shown as mean (*n* = 4) + SD. (**C**,**D**) Wt mice were injected with PBS or LPS (8.3 µg/g body weight) ± ABAH (40 µg/g body weight; injected 2 h before and 5 h after PBS or LPS injection). Cer and SM content of microcapillaries (pooled from three brains per sample) was analyzed by LC-ESI-MS/MS. Data are shown as mean (*n* = 7) + SD. (**E**) Wt mice received a single i.p. injection of PBS or LPS (8.3 µg/g body weight) ± ABAH (40 µg/g body weight; injected twice: 2 h before and 5 h after PBS or LPS injection) and Evans Blue (3% in PBS; 4 µL/g body weight). Twelve hours post treatment, animals were anaesthetized with pentobarbital (150 mg/kg body weight) and transcardially perfused with 25 mL PBS. Brains were removed, frozen in liquid nitrogen, and homogenized. Evans Blue was quantitated spectrophotometrically using an external Evans Blue calibration curve. Results represent mean values (PBS: *n* = 14, PBS+LPS: *n* = 13, PBS+ABAH: *n* = 13, PBS+ABAH+LPS: *n* = 6) ± SD. Significance was calculated by ANOVA, followed by Bonferroni correction. *, *p* ≤ 0.05; ***, *p* ≤ 0.001.

The fact that SL metabolism is under redox control and in turn controls redox signaling [22] prompted us to study the potential involvement of MPO in aberrant SL homeostasis in mouse brain capillaries. Therefore, we used genetic (MPO^−/−^ mice) and pharmacological (the MPO inhibitor ABAH in wt animals) approaches. Of note, the Cer and SM content of brain capillaries isolated either from MPO^−/−^ mice (Figure 2A,B) or from wt mice treated with ABAH (Figure 2C,D) remained unchanged in response to LPS. To test the possibility of pharmacological interference with LPS-induced BBB breakdown, animals received injections of PBS, LPS, or LPS in combination with ABAH followed by administration of Evans Blue (EB). Peripheral LPS increased EB extravasation in this experimental setup from 0.4 to 0.7 µg EB/100 mg brain tissue (control vs. LPS, respectively; Figure 2E). Co-injection of PBS/ABAH was without effect on EB accumulation. Although we observed diminished EB extravasation in LPS/ABAH injected mice as compared to LPS alone (0.56 vs. 0.7 µg EB/100 mg brain, LPS/ABAH vs. LPS), however, this difference did not reach statistical significance (Figure 2E).

**Figure 3:**
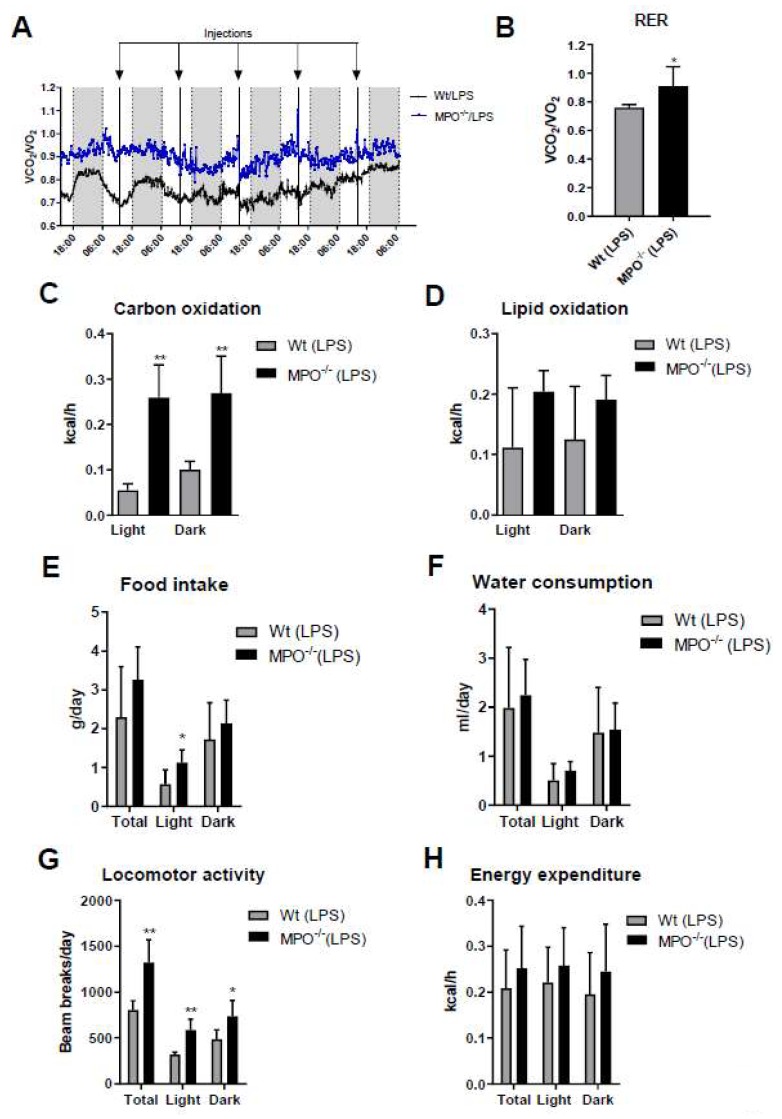
Short term metabolic cage readouts 24 h after first LPS injection. Wt and MPO^−/−^ mice were housed at room temperature in metabolic cages with free access to chow diet and water. Mice were injected daily with LPS (i.p. 0.83 µg/g body weight) for 5 d. (**A**) Total (blue box represents the 24 h period after the first LPS injection) and (**B**) mean respiratory exchange ratio (RER), (**C**) carbohydrate oxidation, (**D**) lipid oxidation, (**E**) daily food intake, (**F**) water consumption, (**G**) locomotor activity, and (**H**) energy expenditure were measured for 24 h after the first LPS injection. Data represent means (*n* = 6) + SD. Significance was calculated by student’s unpaired *t*-test, followed by Welch’s correction in case of unequal variances. *, *p* ≤ 0.05; **, *p* < 0.01.

Activation of the immune system during systemic inflammation can induce sickness behavior [23] and forced expression of human MPO in a murine Parkinson’s Disease model exacerbates motor impairment [24]. HOCl scavengers were suggested to modulate sickness/motor behavior in a murine model of inflammation [25]. To study this aspect more directly we monitored the effects of low-grade inflammation on metabolic, physiological, and behavioral activities of wt and MPO^−/−^ animals. Short (24 h post first LPS injection)- and long-term (averaged over the entire 5-days treatment period) readouts of these experiments are displayed in Figure 3 and Figure S2, respectively. The mean respiratory exchange ratio (RER) measured by indirect calorimetry was calculated as the ratio of the amounts of CO_2_ produced and O_2_ consumed. Total RER (Figure 3A, blue box) and mean RER (Figure 3B) were markedly higher in MPO^−/−^ animals, indicating preferential metabolism of carbohydrates over lipid oxidation. In fact, carbohydrate oxidation was 5.7-fold (light cycle) higher in MPO^−/−^ mice (Figure 3C), whereas lipid oxidation was lower (Figure 3D). Food intake and water consumption during the light cycle was significantly higher in MPO^−/−^ mice (Figure 3E,F). Locomotor activity was markedly increased in MPO^−/−^ mice (Figure 3G), while energy expenditure was unaltered (Figure 3H). The corresponding data averaged over the entire 5-day treatment period are shown in Figure S2. Our findings indicate that MPO^−/−^ mice are less affected by LPS-induced sickness behavior as compared to wt animals.

**Figure 4:**
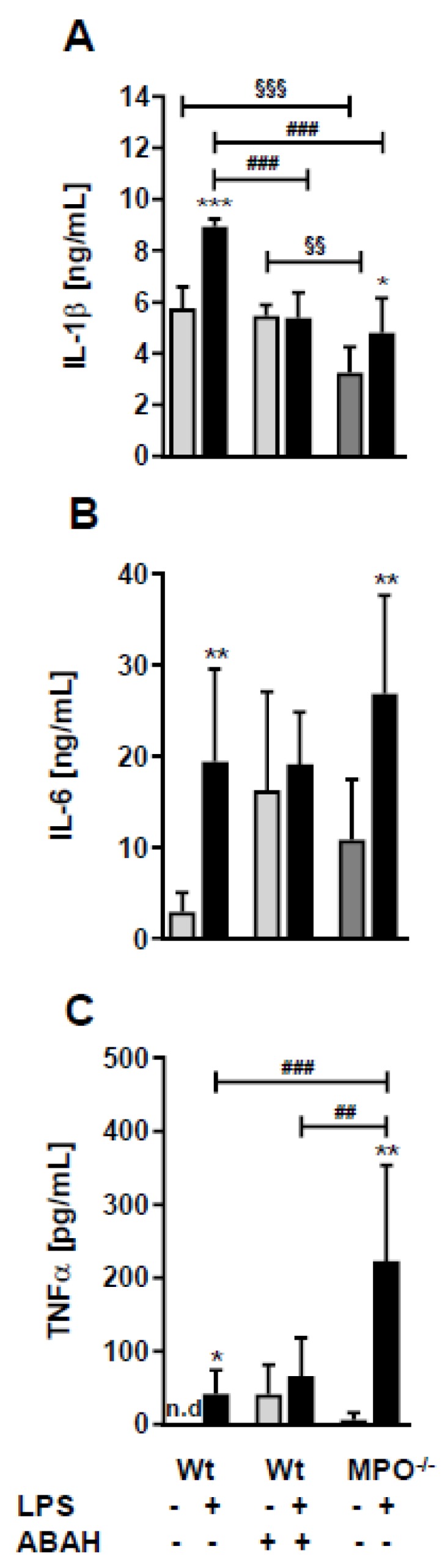
LPS differently affects plasma cytokine levels in wt, ABAH-treated, and MPO^−/−^ mice. Wt and MPO^−/−^ mice received a single i.p. injection of PBS (control) or LPS (8.3 µg/g body weight) ± ABAH (40 µg/g body weight; injected twice: 2 h before and 5 h after PBS or LPS injection). Twelve hours post treatment blood was collected by submandibular puncture and assayed for (**A**) IL-1β, (**B**) IL-6, and (**C**) TNFα concentrations by ELISA. Data are shown as mean (*n* = 7) + SD. Significance was calculated by ANOVA, followed by Bonferroni correction. *, *p* ≤ 0.05; **, ^##^, ^§§^, *p* < 0.01; ^###^, ^§§§^, *p* < 0.001; n.d.: not detected. * wt, PBS vs. LPS; ^##, ###^ wt vs. MPO^−/−^; ^§§^, ^§§§^ wt/PBS vs. wt/PBS/ABAH or MPO^−/−^/PBS.

To elucidate whether the loss or inhibition of MPO affects circulating concentrations of cytokines known to impact sickness behavior and BBB function [23] we quantitated IL-1β, IL-6, and TNFα levels by ELISA. In wt animals, peripheral LPS increased the levels of all three cytokines (Figure 4A–C). Surprisingly, ABAH resulted in already higher baseline concentrations of IL-6 and TNFα, which were not further increased by LPS (Figure 4A–C). Wt, Wild type.

**Figure 5:**
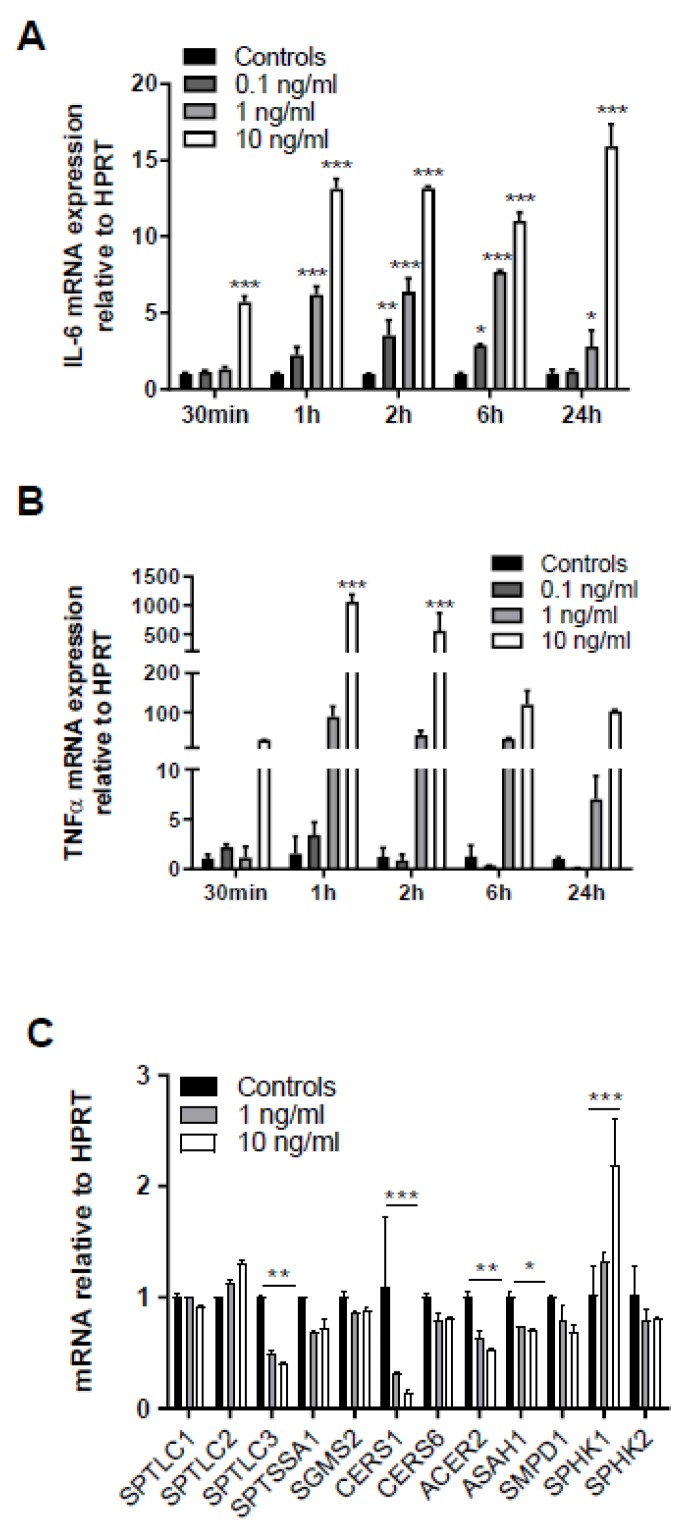
Exogenous TNFα increases in cytokine synthesis and regulates expression of genes involved in sphingolipid metabolism. Serum-starved human brain endothelial (hCMEC/D3) cells were treated with the indicated rTNFα concentrations (0.1–10 ng) for the indicated times and gene expression of (**A**) *IL-6* and (**B**) *TNFα* was evaluated by qPCR analysis. Hypoxanthine-guanine phosphoribosyltransferase (*HPRT*) was used as housekeeping gene. Data are shown as mean (*n* = 4) + SD. (**C**) hCMEC/D3 cells were incubated with 1 or 10 ng/mL TNFα for 2 h and expression of serine palmitoyltransferase 1-3 (*SPTLC1-3*), serine palmitoyltransferase small subunit A (*SPTSSA1*), sphingomyelin synthase 2 (SGMS2), ceramide synthase 1 and 6 (CerS1 and CerS6), alkaline ceramidase 2 (*ACER2*), acid ceramidase (*ASAH1*), sphingomyelinase 1 (*SMPD1*), and sphingosine kinase 1 and 2 (*SPHK1* and *2*) was evaluated by qPCR analysis. Data are shown as mean (*n* = 3) + SD. Expression was calculated using the 2^−dd*C*t^ method. *, *p* ≤ 0.05; **, *p* < 0.01; ***, *p* < 0.001. Significance was calculated by ANOVA, followed by Bonferroni correction.

One of the signaling pathways known to contribute to sepsis-induced BBB dysfunction is triggered by binding of systemic TNFα to the neurovasculature leading to induction of cyto-/chemokine synthesis that causes BBB disruption [26,27]. In line, treatment of human hCMEC/D3 cells with recombinant TNFα (rTNF) upregulated *IL-6* mRNA in a time- and concentration-dependent manner (Figure 5A). Upregulation of endogenous *TNFα* with 1000-fold mRNA induction was even more pronounced after 1 h in response to 10 ng/mL rTNFα (Figure 5B). Thus, these experiments support the notion that extracellular rTNFα can induce a vicious cycle of endogenous cytokine synthesis in BMVEC. qPCR analyses revealed that serine palmitoyltransferase 3 (*SPTLC3*), ceramide synthase 1 (*CERS1*), alkaline ceramidase 2 (*ACER2*), and acid ceramidase (*ASAH1*) were downregulated, while sphingosine kinase 1 (*SPHK1*) gene expression was upregulated in response to the highest TNFα concentration (Figure 5C).

**Figure 6:**
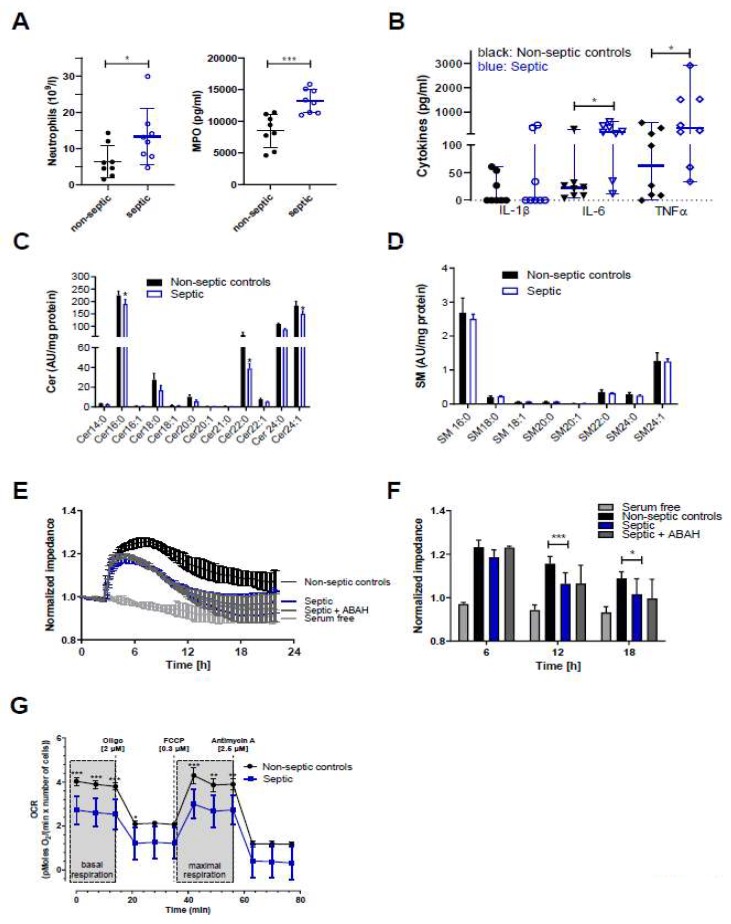
Samples from sepsis patients contain higher neutrophil counts and higher concentrations of MPO, IL-6, and TNFα, and disrupt Cer homeostasis, barrier function, and mitochondrial respiration of hCMEC/D3 cells. (**A**) Blood or serum samples obtained from non-septic and septic patients were assayed for neutrophil counts and MPO. Results are presented as mean ± SD (*n* = 8 per group). (**B**) Cytokine concentrations in serum samples obtained from non-septic and septic patients. Results are shown as median ± 95% CI (*n* = 8 per group). Significance was calculated by Mann-Whitney test. (**C**,**D**) Serum-starved hCMEC/D3 cells were treated with serum from non-septic or septic patients for 12 h. Cer and SM species were measured by LC-ESI-MS/MS. The values obtained were normalized to the internal standard and protein content (arbitrary units (AU)/mg protein). Results are shown as mean (*n* = 4) + SD. (**E**) hCMEC/D3 cells were plated on collagen-coated gold microelectrodes, cultured to confluence, and serum-starved overnight. Impedance of cell monolayers was continuously monitored at 4 kHz (*n* = 2, in duplicate). After stabilization, serum from non-septic or septic patients was added to the cells. Serum free media served as controls. (**F**) Statistical evaluation of normalized impedance values of hCMEC/D3 cells cultured in the presence of serum from non-septic or septic (±ABAH; 100 µM) patients (*n* = 8 per group). Results are shown as mean + SD. (**G**) hCMEC/D3 cells (50.000 cells/well) were cultured with sera (20%) from non-septic (*n* = 4) or septic patients (*n* = 4) and subsequently oxygen consumption rate (OCR) was analyzed in the presence of 25 mM glucose and 2 mM L-glutamine. Cells were treated with 2 μM oligomycin (oligo), 0.3 μM carbonyl cyanide-4-(trifluoromethoxy)phenylhydrazone (FCCP), and 2.5 μM antimycin A. Values were normalized to the protein content. Data are presented as mean values ± SD of sextuplicate determinations. *, *p* ≤ 0.05; **, *p* < 0.01; ***, *p* < 0.001. Significance was calculated by ANOVA, followed by Bonferroni correction.

To gain insight into the effects of septic conditions on hCMEC/D3 cells we initiated a small observational study utilizing serum samples from septic and non-septic patients admitted to the intensive care unit (ICU, sepsis was diagnosed as outlined in Materials and Methods). Serum samples of sepsis patients (as compared to non-septic patients) contained higher neutrophil counts (13.3 × 10^9^ vs. 6.5 × 10^9^ cells/l; Figure 6A, left panel) and higher concentrations of MPO (13.1 vs. 8.8 ng protein/mL; Figure 6A, right panel), IL-6 (188 vs. 23 pg/mL; Figure 6B), and TNFα (348 vs. 63 pg/mL). IL-1β concentrations were below the limit of detection in five samples in both groups (resulting in a median of zero, Figure 6B).

Since proinflammatory cytokines, in particular TNFα, are potent modulators of SL homeostasis we analyzed the Cer and SM content of hCMEC/D3 cells incubated in the presence of non-septic and septic serum samples. In accordance with our in vivo findings in the murine endotoxemia model, LC-MS/MS analyses revealed significantly reduced Cer16:0, Cer22:0, and Cer24:1 content in hCMEC/D3 cells cultured in the presence of sera from sepsis patients (Figure 6C) while SM species remained unaffected when compared to non-septic samples (Figure 6D). Real-time monitoring of hCMEC/D3 barrier function demonstrated that cells cultured in the presence of serum obtained from sepsis patients developed significantly lower transendothelial impedance as compared to non-septic serum (Figure 6E,F). No effects were observed at 64 kHz, being indicative of intact cell monolayers (data not shown). However, despite higher MPO content in serum of sepsis patients, the addition of ABAH resulted only in a marginal increase of impedance values at the 6 h time point (Figure 6F) and was without effect at the 12 and 24 h time points. Inflammatory conditions can impair BBB integrity through induction of a mitochondrial crisis [28]. In line, measuring oxygen consumption rate (OCR) revealed that septic serum samples significantly attenuated basal and maximal respiration of hCMEC/D3 cells (Figure 6G), supporting the concept that inflammatory mediators interfere with BMVEC function.

## 3. Discussion

The present study provides evidence that endotoxemia affects FA, Cer, and SM homeostasis in isolated mouse brain microvessels (Figure 1). Pharmacological or genetic interference with MPO activity showed benefits by maintaining SL homeostasis in response to peripheral LPS. This is reminiscent of what was found in other CNS-related disease models: Inhibition of MPO reduces brain damage in a murine stroke model [29], reduces oxidative stress-induced neuronal damage [30], ameliorates experimental autoimmune encephalomyelitis (EAE) disease severity [31], improves inflammatory parameters in 6-hydroxydopamine induced Parkinsonism [32], and increases neurogenesis after ischemic stroke in mice [33]. Antagonism of MPO activity by ABAH as well as genetic deletion of MPO resulted in improved the outcome in the transient middle cerebral artery occlusion mouse model [34]. MPO^−/−^ mice show better functional recovery after spinal cord injury [35]. Moreover, LPS-induced BBB dysfunction and barrier dysfunction of primary BMVEC was rescued by ABAH treatment [36]. During the present study ABAH slightly diminished LPS-induced EB accumulation in brain (Figure 2E), this effect was, however, not statistically significant. This is probably due to some limitations related to our experimental setup since we did not quantitate circulating dye concentrations. In patients, elevated circulating MPO levels were associated with small vessel-related stroke risk [37]. The Ford group has previously reported that 2-chloro FA formed via HOCl-mediated attack of plasmalogens contribute to pulmonary endothelial injury and have prognostic utility in sepsis-associated acute respiratory distress syndrome [38]. Therefore, the pharmacological benefit for MPO in preclinical settings of neurological disease models including inflammation-induced BBB dysfunction is following the ‘inhibit ROS toxifiers’ concept.

The production of reactive species and proinflammatory cytokines by neutrophils at sites distant from the initial infection suggested that this cell type contributes to organ damage in sepsis [39]. Indeed, Carr and colleagues showed that circulating MPO is elevated in patients with septic shock and associated with organ failure and mortality in critically ill patients despite comparable neutrophil counts in septic shock and non-septic patients [40]. On the other hand, in human sepsis patients diminished MPO expression was identified as the best predictor to identify a subgroup of patients at high risk of death [41]. Reber and colleagues reported the development of a mouse model that enables specific diphteria toxin-induced neutrophil ablation [42]. These authors demonstrated that neutrophils protect the host from LPS-induced lethal inflammation and showed that MPO plays a major role in this protective pathway by adoptive transfer experiments. In contrast to data obtained during the present study where ABAH ameliorated aberrant SL homeostasis in vivo Reber and colleagues reported ABAH-induced mortality in response to LPS-induced inflammation [42]. Currently, the reason(s) for these contradictory findings are still unclear could be, however, due to different LPS and ABAH concentrations used during the present study.

Multifactorial mechanisms that drive aberrant SL homeostasis in brain microcapillaries under inflammatory/septic conditions can be envisaged. Endothelial cells express the enzymatic machinery necessary for de novo FA synthesis. LPS-induced endotoxemia suppresses FA synthase mRNA levels and de novo FA synthesis in vivo [43,44]. We observed reduced total FA content in brain capillaries isolated from endotoxin-treated mice (Figure 1A). This is most probably a reflection of decreased lipolysis and a subsequent decrease of FA import into microcapillaries due to decreased lipoprotein lipase activity and expression of the FA transporter CD36 under inflammatory conditions [45]. Substrate limitation due to reduced availability of palmitic acid for the first and rate limiting step of SL synthesis is a reasonable explanation for decreased Cer and SM concentrations in brain capillaries of LPS-treated wt mice (Figure 1B,C). A similar decrease was observed for several lyso-phosphatidylcholine species in human LPS-treated BMVEC in an organ-on-chip model [46]. SL homeostasis is under control of inflammatory mediators that affect the activity of several enzymes within this metabolic pathway. TNFα stimulates SL metabolic enzymes including sphingomyelinases, ceramidases, and SPHK1 [6,47]. Vutukuri and colleagues demonstrated that endotoxemia increases the Cer concentrations in mouse serum (but not in brain or brain capillaries) while S1P concentrations in serum, whole brain and in brain capillaries are decreased [11]. In brain capillaries, these observations were ascribed to transcriptional and translational upregulation of S1P phosphatase as well as lipid phosphate phosphatase 1, and to downregulation of SPHK2 [11]. In contrast, we observed lower concentrations of Cer and SM species in brain capillaries which might be due to higher LPS concentrations used in the present study (8.3 vs. 4 µg/g body weight).

The ‘inhibit ROS toxifier’ concept holds promise to preserve SL homeostasis in brain capillaries for several reasons: i) sphingomyelinases and ceramidases are regulated by stress signaling [22] that might be attenuated in the absence of MPO activity, ii) sphingomyelinases are activated by ROS and reactive nitrogen species, and iii) HOCl generated by the MPO-H_2_O_2_-Cl^−^ system oxidatively modifies lipids including plasmalogens [38,48,49,50] and SL [19,20,51].

Higher food intake and locomotor activity of LPS-treated MPO^−/−^ animals compared to wt mice (Figure 3) indicate less pronounced sickness behavior in mice lacking MPO. Although this is surprising due to the higher LPS-induced TNFα concentrations observed in MPO^−/−^ mice (Figure 4), which might be a result of increased TNFα release by activated MPO^−/−^ neutrophils [52]. On the other hand, reduced sickness behavior might be due to defective HOCl production and the absence of MPO-derived chlorinated lipids. Whether or not these lipids could induce comparable behavioral deficits as reported for chlorinated dopamine [53] is currently unknown. However, intrastriatal administration of chlorodopamine resulted in loss of dopaminergic neurons and decreased locomotor activity in mice in a manner similar as observed for the Parkinsonian toxin MPP^+^ [53]. Selective serotonin reuptake inhibitors ameliorated LPS-induced sickness behavior in wt mice [25], a potential result of HOCl scavenging by serotonin which is a high-affinity substrate for MPO-mediated oxidation [25]. The fact that brains of transgenic APP/PS1 mice contain far higher HOCl concentrations as compared to wt littermates makes it conceivable that MPO derived oxidants have the potential to contribute to induce behavioral and cognitive deficits [54].

TNFα plays a pivotal role in sepsis-mediated organ dysfunction and induces cerebral edema via TNF receptor 1-mediated pathways in a mouse model [55]. In line, our results demonstrate that exogenously supplied TNFα increases mRNA levels of *TNFα* and *IL-6* in hCMEC/D3 cells (Figure 5A,B), indicating a potentially vicious cycle in the neurovasculature as both cytokines are able to induce BBB dysfunction in vitro [56] and in vivo [57]. TNFα also affects expression of key genes centrally involved in SL homeostasis (Figure 5C). Downregulation of SPTLC3 indicates decreased synthetic flux through 3-ketodihydrosphingosine, the first step in SL biosynthesis ([6]; Figure S1). Reduced mRNA expression of CerS1, which is responsible for the synthesis of C18 Cer and expressed in the mouse brain and spinal cord [58] might explain the lower concentration of these species in brain capillaries isolated from LPS-treated mice. Of note, CerS6, catalyzing Cer16:0 synthesis [58] was decreased in hCMEC/D3 cells treated with TNFα. Accordingly we observed reduced Cer16:0 concentrations in rTNFα-exposed hCMEC/D3 cells and in brain capillaries of LPS-treated wt mice. SL composition in murine brain capillaries and human hCMEC/D3 cells was substantially different (Figure 1B,C and Figure 6C,D). In capillaries Cer18:0 and SM16:0/SM18:0 represent the most abundant species while in hCMECD3 cells Cer16:0 and SM16:0 are the main representatives. These findings might be a result of species-related differences as reported for molecular Cer composition of human prefrontal cortex and murine forebrain [59]. Brain capillary SPHK1 is upregulated in LPS-treated mice [11]. This adaptive mechanism plays an important role during TNFα-mediated NF-kB activation results in SPHK1-dependent S1P formation, S1P binding to TRAF2, and subsequent induction of cytoprotective pathways [60].

Cytokines, matrix metalloproteinases, and/or reactive species increase BBB permeability during the inflammatory response [61]. In septic samples, we observed higher neutrophil counts, as well as higher MPO, IL-6, and TNFα concentrations (Figure 6A,B). In terms of MPO content these findings are in line with a recent report [40], however, in contrast to the present study the authors reported comparable neutrophil numbers in sepsis and non-sepsis samples. In response to septic serum supplementation barrier function was reduced but not recovered in the presence of ABAH (Figure 6E,F). This might be a result of the higher cytokine content in septic serum or insufficient H_2_O_2_ generation by hCMEC/D3 cells needed as co-substrate for HOCl generation via MPO. Seahorse measurements revealed reduced basal and maximal respiration of cells cultured in the presence of sepsis serum (Figure 6G). Mitochondrial dysfunction is a key contributor to LPS-induced exacerbation of BBB dysfunction and stroke outcome in a murine model [28]. Since mitochondrial function depends on SL homeostasis [62], the drop in OCR might result from decreased Cer content (Figure 6C) in hCMEC/D3 cells cultured in the presence of plasma from sepsis patients. It is plausible that decreased CERS1 mRNA expression (Figure 6C) and a concomitant downregulation of Hsp27/CERS1-dependent pathways could account for decreased mitochondrial Cer levels. If occurring (not addressed experimentally in this study), a reduction in mitochondrial Cer concentrations likely induces structural and functional changes in mitochondria as observed in a model of Charcot-Marie-Tooth disease, the most commonly inherited neurological disorder [63].

We conclude that MPO and inflammatory mediators affect SL homeostasis in murine brain microcapillaries in vivo and human brain endothelial cells in vitro. Pharmacological inhibition of the ROS toxifier MPO holds promise to interfere with aberrant SL homeostasis in brain capillaries and to partially rescue BBB dysfunction under septic conditions in vivo.

## 4. Materials and Methods

### 4.1. Materials

Cell culture supplies were from Thermo Fisher Scientific (Waltham, MA, USA) and Sigma-Aldrich (St. Louis, MO, USA). Plastic ware for cell culture was obtained from Costar (Vienna, Austria) or VWR (Vienna, Austria). Seahorse XF96 polystyrene D3 cell culture microplates were from Seahorse Bioscience^®^ (Agilent; Santa Clara, CA, USA). Human ELISA kits were from ImmunoTools (Friesoythe, Germany). Mouse ELISA kits were from Peprotech (Rocky Hill, NJ, USA). LPS from *E. coli* (O111:B4), pentobarbital sodium salt, ABAH, and Evans Blue (EB) were from Sigma-Aldrich (St. Louis, MO, USA). Human recombinant TNFα (rTNFα) was from Thermo Fisher (Vienna, Austria). Electrical cell-substrate impedance sensing (ECIS) electrode arrays (8W10E+) were from Ibidi (Martinsried, Germany). Sera from non-sepsis or sepsis patients were obtained from the Department of Internal Medicine, ICU, Medical University of Graz. Kits that were used for quantitative real-time PCR (qPCR) analysis were from QIAGEN (Hilden, Germany) or Applied Biosystems (Foster City, CA, USA). Primers were ordered from QIAGEN.

### 4.2. Animals

MPO^−/−^ mice (B6.129X1-Mpo^tm1Lus^/J) were obtained from the Jackson Laboratory (Bar Harbor, ME, USA). Genotyping was performed from ear tags using standard PCR protocols. C57BL/6 mice were obtained from the Department of Laboratory Animal Science (Himberg, Austria). All animals were kept on a 12 h light/dark cycle with free access to food and water. Animal experiments were approved by the Austrian Federal Ministry of Science, Research, and Economy, Division of Genetic Engineering and Animal Experiments (BMBWF-66.010/0067-V/3b/2018). The Austrian Federal Ministry of Education, Science and Research, Division of Genetic Engineering and Animal Experiments approved the animal experiments (06.07.2018; BMWF-66.010/0067-V/3b/2018). The Ethics Committee of the Medical University of Graz approved this observational clinical study (Dec 27th 2018; #30-258 ex 17/18), and all patients provided written informed consent.

### 4.3. Evans Blue (EB) Extravasation

Wt and MPO^−/−^ mice (8–12 weeks, 20–30 g) were injected i.p. with PBS (pH 7.4) or LPS (8.3 µg in PBS/g body weight) ± MPO inhibitor ABAH (40 µg/g body weight). Changes in vascular permeability during systemic inflammation were determined by i.p. injection of 3% EB in PBS (4 µL/g body weight) at treatment start. After 12 h mice were anesthetized with 150 mg/kg pentobarbital and transcardially perfused with PBS. Subsequently, brains were removed, weighed, and homogenized in dimethylformamide (DMF). After extraction for 1 h on a rotating wheel, samples were centrifuged at 21,500× *g* for 15 min and EB was quantified spectrophotometrically at 620 nm using an external calibration curve. Due to known limitations of the EB method (e.g., spectral shifts of EB absorption in the presence of proteins, leakage of free dye, or toxicity when injected at very high concentrations) we used the dye at average concentration, extracted the dye with DMF, always prepared external EB standards immediately prior to measurements, and do not claim that EB accumulation equals albumin extravasation into the brain. Time points < 24 h post LPS (8.3 µg/g body weight) were chosen to minimize animal suffering according to the 3R principles (in our experience animal survival at 16 h is 100% while mortality significantly increases at time points > 18 h).

### 4.4. Brain Capillary Isolation

Mouse brain microcapillaries were isolated by density-gradient centrifugation, as described previously [64]. Briefly, a mouse brain was immersed in ice-cold PBS and the cerebellum, meninges, brainstem, and large blood vessels were removed. The cortex was homogenized in ice-cold DMEM (Gibco) containing 10% (*v*/*v*) fetal calf serum (FCS) in a Dounce homogenizer. The homogenate was centrifuged at 500× *g* for 10 min at 4 °C and the supernatant was discarded. The pellet was homogenized in DMEM containing 10% FCS and passed through a 100 µm mesh nylon filter. The filtrate was passed again through a 40 µm mesh nylon filter. The fraction retained on the 40 µm filter was collected, suspended in ice-cold DMEM containing 10% FCS and centrifuged at 12,000× *g* for 45 min at 4 °C. The supernatant was discarded and the pellet containing the microcapillaries was resuspended in 500 µL erythrocyte lysis buffer. After 2 min, 500 µL PBS were added to stop the reaction and the microcapillaries were again spun down at 12,000× *g* for 20 min at 4 °C. The supernatant was discarded and the pellet stored at −80 °C.

### 4.5. Indirect Calorimetry (Energy Expenditure, Locomotor Activity)

MPO^−/−^ and wt mice were individually housed in PhenoMaster cages (TSE Systems, Bad Homburg, Germany) for measuring energy intake- and expenditure-related data over 5 days and injected with LPS (0.83 µg in PBS/g body weight) every 24 h. This chronic low-dose endotoxemia model was chosen to be able to monitor animal behavior over an extended period of time (in the acute high-dose LPS model used in the experimental paradigm described above animal suffering increases at time points >18 h post LPS application). The cage system included photobeam-based activity monitoring that records ambulatory movements. An indirect gas calorimetry system simultaneously measured oxygen consumption (VO_2_), carbon dioxide production (VCO_2_), respiratory exchange ratio (RER), and food and water intake.

### 4.6. Human Blood Samples

Patients were recruited from the ICU of the Department of Internal Medicine at the Medical University of Graz. The Ethics Committee of the Medical University of Graz approved the prospective study protocol (#30-258 ex17/18), and all patients provided a written informed consent. Critically-ill patients with sepsis were defined by sepsis-3 criteria with a suspected infection and a rise in sequential organ failure assessment (SOFA) score ≥ 2 [65]. Comfort terminal care, pregnancy, and age > 100 years were exclusion criteria. Critically ill patients without infection served as controls. Immediately after admission to the ICU blood samples (4 mL) were collected and serum was isolated and stored at −80 °C. Neutrophils were immediately counted using an automatic blood cell counter (Sysmex, Kobe, Japan).

### 4.7. Cell Culture

hCMEC/D3 cells [66] were cultured in rat-collagen-coated 75 cm^2^ flasks in Earl’s salts-containing Medium 199 supplemented with 10% (*v*/*v*) FCS, 1% (*v*/*v*) streptomycin/penicillin, 1% (*v*/*v*) chemically defined lipid concentrate (ThermoFisher), 1% (*v*/*v*) HEPES buffer, 1.4 μM hydrocortisone, 5 μg/mL ascorbic acid, and 1 ng/mL bovine fibroblast growth factor at 37 °C (5% CO_2_) until confluence. Prior to experiments, cells were serum-starved overnight in Earls salts-containing M199. Only passages below 38 were used for experiments.

### 4.8. Gas Chromatography (GC)

Mouse brain capillary lipids were extracted twice with chloroform/methanol (2:1, *v*:*v*) and dried under a stream of nitrogen. After addition of the internal standard (pentadecanoic acid), lipids were trans-esterified (1.2 mL toluene and 1 mL boron trifluoride-methanol (20%)) at 110 °C for 1 h. GC analysis of the corresponding FA methyl esters was performed as described [67] and concentrations were quantitated by peak area comparison with the internal standard and normalized to total cell protein concentrations.

### 4.9. Liquid Chromatography-Electrospray Ionization-Mass Spectroscopy (LC-ESI-MS/MS)

Samples were mixed with chloroform/methanol (2:1; *v*/*v*) and Cer17:0 and SM17:0 as internal standards (Avanti Polar Lipids, Alabaster, USA). For SL analysis, lipid extracts were subjected to a mild alkaline hydrolysis step by adding 400 µL 1 M NaOH in chloroform/methanol/H_2_O (16:16:5, *v*/*v*/*v*) and incubated for 45 min at room temperature. Samples were neutralized (150 µL of 1 M acetic acid, 400 µL of 0.5 M EDTA), and 1 mL CHCl_3_ was added. Samples were vortexed, centrifuged, and the upper aqueous layer was removed. After a second washing step (700 µL H_2_O), the organic layer was dried under a gentle stream of nitrogen. Lipids were redissolved in 120 µL 2-propanol/methanol/water (7/2.5/1, *v*/*v*/*v*) for UPLC-MS analysis. Chromatographic separation was modified after Knittelfelder et al. [68] using an ACQUITY-UPLC system (Waters Corporation), equipped with a Luna omega C18 column (2.1 × 50 mm, 1.6 µm; Phenomenex) starting a 20 min linear gradient with 80% solvent A (MeOH/H2O, 1:1, *v*/*v*; 10 mM ammonium acetate, 0,1% formic acid, 8 µM phosphoric acid). The column compartment was kept at 50 °C. A EVOQ Elite™ triple quadrupole mass spectrometer (Bruker) equipped with an ESI source was used for detection of lipids in positive ionization mode. Lipid species were analyzed by selected reaction monitoring (Cer, [M+H]+ to *m*/*z* 264.3, 22 eV, 60 ms; SM, [M+H]+ to *m*/*z* 184.1, 20 eV, 40 ms; resolution 0.7 Q1/Q3). Data acquisition was done by MS Workstation (Bruker). Data were normalized for recovery, extraction-, and ionization efficacy by calculating analyte/IS ratios (arbitrary units; AU) and expressed as AU/mg protein.

### 4.10. ELISA

Wt and MPO^−/−^ mice were injected i.p. with 200 µL PBS (controls) or 200 µL LPS (8.3 µg in PBS/g body weight ± MPO inhibitor ABAH (40 µg/g body weight). After 12 h, blood was collected by submandibular puncture. Serum IL-1β, IL-6, and TNFα concentrations were quantitated using mouse ELISAs (Peprotech, Vienna, Austria). For human serum samples ELISAs from ImmunoTools (Friesoythe; Germany) were used. Serum MPO concentrations were determined using a commercial ELISA kit (Abcam, Cambridge, UK). The assays were performed according to manufacturer’s instructions. The concentrations of the cytokines were determined using the external standard curve.

### 4.11. Electrical Cell-Substrate Impedance Sensing (ECIS)

Barrier function was determined by impedance monitoring with an (ECIS Z system, Applied Biophysics, Troy, NY, USA). hCMEC/D3 cells were seeded on collagen-coated gold electrodes of 8W10E+ arrays, cultured until confluence and serum-starved overnight prior to the experiment. Impedance was recorded in real time at 1 min intervals at 4 kHz (barrier function) and 64 kHz (integrity of cell monolayer). Measurements were performed in the presence of 20% (*v*/*v*) serum from non-sepsis or sepsis (±ABAH, 100 µM) patients as indicated. Despite the advantage of resembling a human system, this cell line develops lower transendothelial electrical resistance values and displays higher permeability as compared to primary culture models [69].

### 4.12. Mitochondrial Respiration Measurement

Fifty thousand hCMEC/D3 cells were seeded per well in an Agilent Seahorse XF96 Cell Culture Microplate coated with Cell-Tak™ according to manufacturer’s protocol. After 24 h serum-starvation, cells were washed and preincubated for 30 min in XF assay medium supplemented with sodium pyruvate (1 mM) with or without glutamine (2 mM) and glucose (25 mM) at 37 °C in a non-CO_2_ environment. The oxygen consumption rate (OCR) was subsequently measured every 7 min using an XF96 extracellular flux analyzer (Seahorse Bioscience, North Billerica, MA, USA). A standard protocol with 15 min basal measurement followed by 2 µM oligomycin (ATP synthase inhibition), addition of 1 µM carbonyl cyanide p-trifluoromethoxy-phenylhydrazone (FCCP; proton gradient disruption), and 2.5 µM antimycin A (complexIII inhibition) was applied. Oxygen consumption was normalized to cell number (pmol O_2_/min × number of cells). Samples were measured in sextuplicate.

### 4.13. qPCR Analysis

Serum-starved hCMEC/d3 cells were stimulated with rTNFα (0.1 ng/mL, 1 ng/mL, 10 ng/mL) followed by total RNA isolation using the RNeasy Mini Kit (Qiagen, Hilden, Germany). RNA was quantitated by NanoDrop measurements (Thermo Fisher Scientific; Waltham, MA, USA) and reverse-transcribed using the high-capacity cDNA reverse transcription kit (Applied Biosystems; Foster City, CA, USA). qPCR was carried out on an Applied Biosystems 7900HT Fast Real-Time PCR system using Quantifast^TM^ SYBR Green PCR. Relative gene expression was normalized to hypoxanthine-guanine phosphoribosyltransferase (HPRT). Expression profiles and associated statistical parameters were analyzed by the 2^−dd*C*t^ method.

### 4.14. Statistical Analysis

All experiments were performed using the appropriate number of replicates per experimental group that is indicated in the respective figure legend. Statistical analyses were performed using the GraphPad Prism version 5 for Mac (GraphPad Software, Inc., San Diego, CA, USA). Testing for normal distribution was performed by the Kolmogorov-Smirnov test (in case of non-normal distribution significance was calculated by Mann-Whitney test). Data obtained from independent measurements were analyzed by one-way ANOVA followed by Bonferroni’s post hoc test. For the analysis of two experimental groups, the unpaired Student’s *t*-test was used.

## Supplementary Materials

Supplementary materials can be found at https://www.mdpi.com/1422-0067/21/3/1143/s1.

## Abbreviations

ABAH	4-aminobenzoic acid hydrazide
ACER	Alkaline ceramidase
BBB	Blood–brain barrier
BMVEC	Brain microvascular endothelial cells
Cer	Ceramide
CerS	Ceramide synthase
CNS	Central nervous system
EAE	Experimental autoimmune encephalomyelitis
ECIS	Electric cell-substrate impedance sensing
FA	Fatty acid
FCCP	Carbonyl cyanide-4-(trifluoromethoxy)phenylhydrazone
HOCl	Hypochlorous acid
HPRT	Hypoxanthine-guanine phosphoribosyltransferase
LPP	Lipidphosphate phosphatase
LPS	Lipopolysaccharide
MPO	Myeloperoxidase
OCR	Oxygen consumption rate
RER	Respiratory exchange ratio
ROS	Reactive oxygen species
S1P	Sphingosine-1-phosphate
S1PR	Sphingosine-1-phosphate receptor
SGMS	Phosphatidylcholine:ceramide cholinephosphotransferase
SGPP	Sphingosine-1-phosphate phosphatase
SL	Sphingolipid
SM	Sphingomyelin
SMPD	Sphingomyelin phosphodiesterase
SOFA	Sepsis-associated organ failure
SPHK	Sphingosine kinase
SPT1-3	Serine palmitoyltransferase 1-3 (gene name SPTLC1-3)
SPTSS	Serine palmitoyltransferase small subunit
Wt	Wild-type
