# Supplementary material for: Myeloperoxidase and Septic Conditions Disrupt Sphingolipid Homeostasis in Murine Brain Capillaries In Vivo and Immortalized Human Brain Endothelial Cells In Vitro
Source: Int J Mol Sci. 2020 Feb 9;21(3):1143. doi: 10.3390/ijms21031143 (PMC7037060; doi:10.3390/ijms21031143)
Supplement: Supplementary file 1 [file ijms-21-01143-s001.pdf]

SUPPLEMENTARY INFORMATION

Myeloperoxidase and septic conditions disrupt sphingolipid homeostasis in murine brain capillaries *in vivo* and immortalized human brain endothelial cells *in vitro*

Madeleine Goeritzer[1,2], Eva Bernhart[1], Ioanna Plastira[1], Helga Reicher[1], Christina Leopold[1], Thomas O. Eichmann[2,3,4], Gerald Rechberger[2,3,4], Corina T. Madreiter-Sokolowski[1,5], Juergen Prasch[1], Philipp Eller[6], Wolfgang F. Graier[1], Dagmar Kratky[1,2], Ernst Malle[1], and Wolfgang Sattler[1,2,4*]

[1]Division of Molecular Biology and Biochemistry, Gottfried Schatz Research Center, Medical University of Graz, Graz, Austria

[2]BioTechMed-Graz, Graz, Austria

[3]Institute of Molecular Biosciences, University of Graz, Graz, Austria

[4]Center for Explorative Lipidomics, BioTechMed-Graz, Graz, Austria

[5]Department of Health Sciences and Technology, ETH Zurich, Schwerzenbach, Switzerland

[6]Department of Internal Medicine, Intensive Care Unit, Medical University of Graz, Graz, Austria

Fig. S1

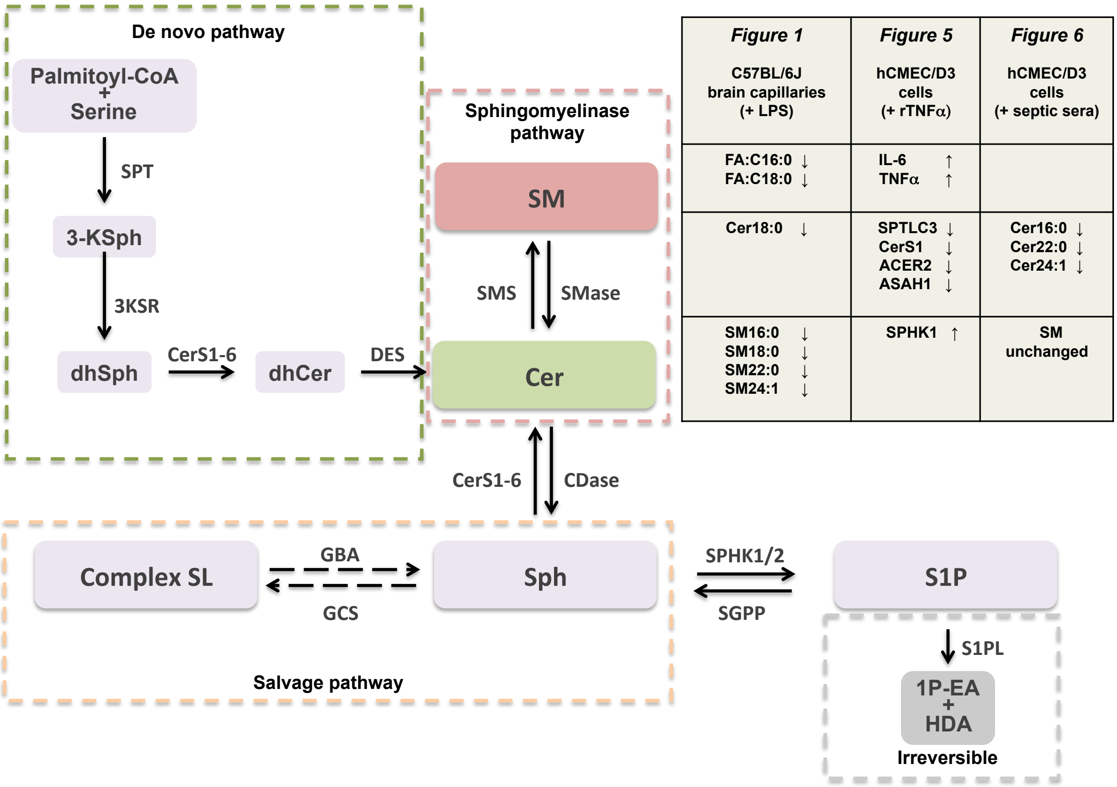

Figure 1<br>C57BL/6J brain capillaries (+ LPS)	*Figure 5*<br>hCMEC/D3 cells (+ rTNFα)	*Figure 6*<br>hCMEC/D3 cells (+ septic sera)
FA:C16:0 ↓<br>FA:C18:0 ↓	IL-6 ↑<br>TNFα ↑	
Cer18:0 ↓	SPTLC3 ↓<br>CerS1 ↓<br>ACER2 ↓<br>ASAH1 ↓	Cer16:0 ↓<br>Cer22:0 ↓<br>Cer24:1 ↓
SM16:0 ↓<br>SM18:0 ↓<br>SM22:0 ↓<br>SM24:1 ↓	SPHK1 ↑	SM unchanged

Fig. S1: Major steps of sphingolipid synthesis/interconversion and alterations observed during the present study.

Ceramide is the central SL metabolite and can be generated by different pathways. De novo SL synthesis (outlined in green box) starts with condensation of palmitoyl-CoA and serine by the serine palmitoyl transferase complex (SPT) resulting in the generation of 3-ketosphinganine (3-KSph). 3-KSph-reductase (3KR) generates dihydrosphingosine (dhSph), which is further converted to dihydroceramides (dhCer) by ceramide synthases (CerS1-6) that show isoform specificity for the transferred acyl residues. dh-Cer desaturase (DES) then reduces dh-Cer to ceramides (Cer).

In the sphingomyelinase pathway Cer can be generated via sphingomyelin (SM) hydrolysis, a step catalyzed by sphingomyelinases (SMase). SM synthesis from CER is facilitated by sphingomyelin synthases (SMS) that use phosphatidylcholine as co-substrate.

Conversion of sphingosine (Sph) to ceramide is catalyzed by CerS1-6 via the Salvage pathway. Cer is metabolized to sphingosine-1-phosphate (S1P) by Sph generation via ceramidases (CDase) and subsequent action of sphingosine kinases (SPHK1 and SPHK2). S1P can be de-phosphorylated to Sph via S1P phosphatases (SGPP). Cleavage of S1P by S1P lyase (S1PL) into fatty aldehydes (e.g. hexadecanal, HDA) and phosphoethanolamine (1P-EA) represent an irreversible step within the metabolic SL cycle. Sph serves as

precursor for complex SL (via glucosylceramide synthase; GCS), and Cer is a precursor for ceramide-1-phosphate synthesis via ceramide kinase (not shown).

The Table inset (right) shows alterations of molecular Cer and SM species in brain capillaries isolated from LPS injected C57BL/6J mice (Fig. 1) and hCMECD3 cells cultured in the presence of septic sera (Fig. 6). hCMECD3 cells supplemented with human recombinant TNFα upregulated gene expression of IL-6 and endogenous TNFα (Fig. 5A,B). Gene expression of the SPTLC3 subunit of SPT, CerS1, alkaline CDase 2 (ACER2), and acid CDase (ASAH1) were downregulated, while SPHK1 was upregulated (Fig. 5C).

Fig. S2

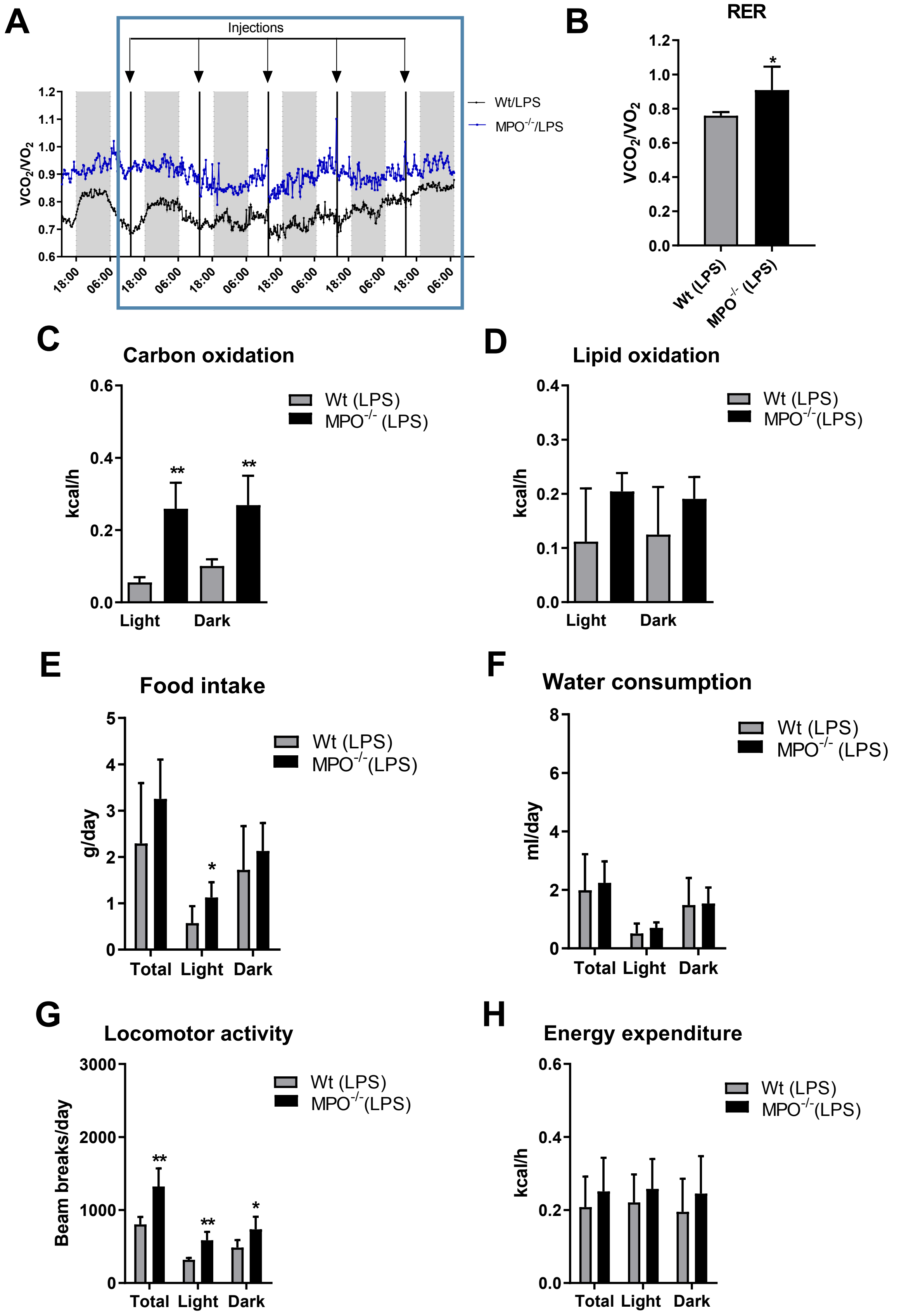
A
Injections
Wt/LPS
MPO-/-/LPS
VCO2/VO2
1.2
1.1
1.0
0.9
0.8
0.7
0.6
18:00
06:00
B
RER
VCO2/VO2
1.2
1.0
0.8
0.6
0.4
0.2
0.0
*
Wt (LPS)
MPO-/- (LPS)
C
Carbon oxidation
kcal/h
0.6
0.4
0.2
0.0
**
**
Wt (LPS)
MPO-/- (LPS)
Light
Dark
D
Lipid oxidation
kcal/h
0.4
0.3
0.2
0.1
0.0
Wt (LPS)
MPO-/-(LPS)
Light
Dark
E
Food intake
g/day
5
4
3
2
1
0
*
Wt (LPS)
MPO-/-(LPS)
Total
Light
Dark
F
Water consumption
ml/day
8
6
4
2
0
Wt (LPS)
MPO-/- (LPS)
Total
Light
Dark
G
Locomotor activity
Beam breaks/day
3000
2000
1000
0
**
**
*
Wt (LPS)
MPO-/-(LPS)
Total
Light
Dark
H
Energy expenditure
kcal/h
0.6
0.4
0.2
0.0
Wt (LPS)
MPO-/-(LPS)
Total
Light
Dark

Fig. S2: Long term metabolic cage readouts (related to Fig. 3).

Wild-type (Wt) and MPO-/- mice were housed at room temperature in metabolic cages with free access to chow diet and water. Mice were injected daily with LPS (i.p. 0.83 µg/g body weight) for 5 days. (A) Total (blue box) and (B) mean respiratory exchange ratio (RER), (C) carbohydrate oxidation, (D) lipid oxidation, (E) daily food intake, (F) water consumption, (G) locomotor activity, and (H) energy expenditure were measured by indirect gas calorimetry. Data represent means (n=6) + SD of the entire observation period.

Significance was calculated by student's unpaired t-test, followed by Welch's correction in case of unequal variances. *, $p \leq 0.05$; **, $p<0.01$.
